# Thiol redox switches regulate the oligomeric state of cyanobacterial Rre1, RpaA and RpaB response regulators

**DOI:** 10.1002/1873-3468.14340

**Published:** 2022-04-11

**Authors:** Iskander M. Ibrahim, Stephen J. L. Rowden, William A. Cramer, Christopher J. Howe, Sujith Puthiyaveetil

**Affiliations:** ^1^ Department of Biochemistry and Center for Plant Biology Purdue University West Lafayette IN USA; ^2^ Department of Biochemistry University of Cambridge UK; ^3^ Department of Biological Sciences Purdue University West Lafayette IN USA

**Keywords:** Hik2, RpaA, RpaB, Rre1, thiol regulation, TrxA

## Abstract

Cyanobacteria employ two‐component sensor‐response regulator systems to monitor and respond to environmental challenges. The response regulators RpaA, RpaB, Rre1 and RppA are integral to circadian clock function and abiotic stress acclimation in cyanobacteria. RpaA, RpaB and Rre1 are known to interact with ferredoxin or thioredoxin, raising the possibility of their thiol regulation. Here, we report that *Synechocystis* sp. PCC 6803 Rre1, RpaA and RpaB exist as higher‐order oligomers under oxidising conditions and that reduced thioredoxin A converts them to monomers. We further show that these response regulators contain redox‐responsive cysteine residues with an *E_m7_* around −300 mV. These findings suggest a direct thiol modulation of the activity of these response regulators, independent of their cognate sensor kinases.

## Graphical abstract

We show thiol‐mediated redox regulation of cyanobacterial response regulators Rre1, RpaA and RpaB, which are involved in circadian clock function and abiotic stress acclimation. Oxidative stress causes these response regulators to oligomerise independently of phosphorylation, adding another layer of regulation to their DNA‐binding activity. Reduced thioredoxin A converts the higher‐order oligomers of these response regulators to monomers.

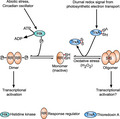

## Abbreviations

**CSK**, chloroplast sensor kinase

***E*_m_**, midpoint potential

***E_m7_***, midpoint potential at pH 7

**FTR**, ferredoxin‐thioredoxin reductase

**H_2_O_2_**, hydrogen peroxide

**Hik**, histidine kinase

**hspA**, heat shock protein A

**LB**, lysogeny broth

**MBP**, maltose‐binding protein

**NTR**, NADPH‐thioredoxin reductase

**PQ**, plastoquinone

**PSI**, photosystem I

**PSII**, photosystem II

**RpaA and RpaB**, regulator of phycobilisome association A and B

**RppA**, regulator of photosynthesis and photopigment‐related gene expression A

**Rre1**, response regulator 1

**SasA**, *Synechococcus* adaptive sensor A

**Trx**, thioredoxin

Two‐component systems allow microorganisms to adapt rapidly to a wide range of environmental conditions through integrated regulation of gene expression. Consisting of a sensor histidine kinase and a response regulator proteins, a two‐component system employs transient phosphorylation and dephosphorylation cycles to convert an environmental stimulus into an appropriate physiological response. A typical cyanobacterial genome encodes multiple two‐component systems. These can vary from as few as five histidine kinases and six response regulators in the genome of *Prochlorococcus* MED4 to as many as 146 histidine kinases and 168 response regulators in the filamentous cyanobacterium *Nostoc punctiforme* [1, 2]. Of these, Response Regulator 1 (Rre1) and Regulator of Phycobilisome Association A and B (RpaA and RpaB) are conserved in all sequenced cyanobacteria. Homologues of Rre1 and RpaB are also found in chloroplasts of certain non‐green algae such as Ycf29 and Ycf27, respectively [3, 4].

Most sensor kinases have just one cognate response regulator partner to which they transmit the environmental information through a phosphotransfer mechanism. Such two‐component systems are usually found as part of the same operon [5]. Some sensor kinases and response regulators, on the other hand, are encoded as orphan proteins. These two‐component signalling elements are frequently far more promiscuous than their chromosomally paired counterparts [6]. Rre1 is an orphan response regulator and, as evidence of its promiscuity, it has been found to work with two sensor kinases – histidine kinases 2 and 34 (Hik2 and Hik34) [7, 8]. Hik2, an orphan sensor kinase, likewise interacts with, and competitively transfers phosphoryl groups to, not only Rre1 but also a second response regulator called Regulator of Photosynthesis and Photopigment‐related gene expression A (RppA) [7, 9]. Analysis of *Synechocystis* sp. PCC 6803 *rre1* mutant revealed that it is required for transcriptional control of salt and heat tolerance genes [10, 11]. Hik2 and its plant orthologue, Chloroplast Sensor Kinase (CSK), were recently shown to sense the redox state of the plastoquinone (PQ) pool *via* an iron‐sulfur cluster [12]. Furthermore, *Arabidopsis* CSK controls photosystem gene expression in a PQ redox‐dependent manner [12, 13, 14, 15]. Taken together, these observations support a role for Hik2‐Rre1 in regulation of photosynthetic gene expression. RppA also likely forms an additional phosphotransfer pathway with the RppB sensor kinase found in the same operon. RppB is a nickel sensor histidine kinase that regulates nickel responsive gene expression in cyanobacteria [16]. In addition to its role in nickel homeostasis, RppA has also been shown to regulate photosynthetic gene transcription in response to the redox state of the PQ pool [17], possibly *via* Hik2 [7].

RpaA and RpaB were first identified as specific regulators of excitation energy transfer from the phycobilisome antennae to reaction centres [3, 18]. RpaA forms a two‐component pair with the *Synechococcus* Adaptive Sensor A (SasA) histidine kinase, which directly interacts with the central circadian clock component KaiC. RpaB forms a two‐component pair with the histidine kinase 33 (Hik33, also known as NblS), and is required for responses to multiple environmental conditions including high light, oxidative stress and high salinity. The circadian rhythm in cyanobacteria is generated by a central oscillator composed of the KaiA, KaiB and KaiC proteins [19], which serves as an endogenous time‐keeping system that allows cyanobacteria to anticipate diurnal changes in environmental conditions. Light entrains the circadian clock in cyanobacteria but not through photoreceptors [20, 21]. The light input is instead derived of the photosynthetic electron transport chain [22]. Together, the SasA‐RpaA and Nbls‐RpaB systems constitute the primary output components of the cyanobacterial circadian clock [23, 24, 25].

In a typical two‐component system, the histidine kinase detects a signal and, upon activation and autophosphorylation, transphosphorylates its cognate response regulator on a conserved aspartate residue. The aspartate phosphorylation creates a high‐energy acyl phosphate in the response regulator receiver domain. This receiver domain modification results in dimerisation or higher‐order oligomerisation of the response regulator, or, in some cases, it modifies the interaction with the effector domain. This results in an appropriate output response – usually a change in gene expression. A few response regulators have been found to be regulated, independently of their cognate sensor kinases, by modifications such as acetylation, serine/threonine phosphorylation or small molecule ligand binding [26, 27, 28]. Indeed, Rre1, RpaA and RpaB have been shown to interact with ferredoxin or thioredoxin and in the case of RpaA and RpaB TrxA is able to reduce them in vitro [23, 24, 25, 29]. This raises the possibility of direct thiol regulation of these response regulators by the redox state of the photosynthetic electron transport chain. In this study, we build on these observations to understand the redox regulatory mechanism of these response regulators in detail. We also included RppA in our analysis given its interaction with Hik2 and its role in photosynthetic gene regulation.

## Materials and methods

### Construction of recombinant plasmids

All clones used here are as described in [7]. Coding sequences for the full‐length Rre1 (*slr1783*), RppA (*sll0797*), RpaA (*sll0797*), RpaB (*slr0947*), and TrxA (*slr0623*) were amplified from *Synechocystis* sp. PCC 6803 genomic DNA using the primer pairs listed in Table 1. PCR products were digested with *KpnI* and *XhoI* endonucleases (New England BioLabs) and cloned into pETG‐41A (EMBL) expression vector. The identities of the recombinant clones were confirmed by sequencing (results not shown).

**Table 1:** Primer pairs used for cloning *Rre1*, *RpaA*, *RpaB, RppA and TrxA*. Sequences in lower case are restriction site overhangs

– Rre1F_MBP (cloned into pETG‐41A)
Forward: GCGCGCggtaccGTGGGCTTGAGTTTGCTG
Reverse: GCGGCGctcgagCTAGACGATCGCCTCCAATTC
– RppAF_MBP (cloned into pETG‐41A)
Forward: GCGCGCggtaccCGAATTTTGCTGGTGGAA
Reverse: GCGGCGctcgagCTACAGTCTTGCTAATAGCTC
– RpaAF_MBP (cloned into pETG‐41A)
Forward GCGCggtaccATGCCTCGAATACTGATC
Reverse: GCGCGCctcgagCTACGTTGGACTACCGCC
– RpaBF_MBP (cloned into pETG‐41A)
Forward: GCGCGCggtaccGTGGTCGATGACGAGGCC
Reverse: GCGGCGctcgagCTAGATTCTAGCTTCCAATTC
– TrxA_MBP (cloned into pETG‐41A)
Forward: GCGCGCggtaccAGTGCTACCCCTCAA
Reverse: TGCGGCGctcgagAAGATATTTTTCTAGGGT

### Expression and purification of recombinant proteins

The recombinant plasmid constructs were introduced into BL21(DE3) chemically competent cells (Stratagene) by transformation. Colonies that grew on agar selection plates were used to inoculate overnight starter cultures in 10 mL Lysogeny Broth (LB) supplemented with 100 µg·mL^−1^ ampicillin. The overnight cultures were diluted to 1 : 100 in l L LB medium and grown at 37 °C until an optical density at 600 nm of 0.55 was reached. Recombinant protein over‐expression was initiated by adding IPTG at a final concentration of 0.5 mm. The cultures were subsequently grown at 16 °C for further 16 h. Cells were harvested by centrifugation at 9000 × ***g*** for 10 min and the pellets were re‐suspended in lysis buffer (20 mm Tris–HCl pH 8, 300 mm NaCl, 25 mm imidazole and 1 mm PMSF) and lysed with an EmulsiFlex‐C3 homogeniser (Avestin). The lysate was separated by centrifugation at 39 000 × ***g*** for 20 min at 4 °C. The supernatant was applied to a Ni^2+^ affinity column (Cytiva, Uppsala, Sweden) and purified according to the manufacturer’s instructions. The purified proteins were buffer exchanged with a PD‐10 column into a buffer medium containing 0.1 mm NaCl and 0.1 mm HEPES (pH 8.0).

### Redox treatment

Aliquots of 2.5 µm desalted full‐length recombinant Rre1, RpaA, RpaB and RppA proteins were incubated with a final concentration of 2 mm cysteine‐specific oxidant diamide, H_2_O_2_ or with the reductant DTT for 30 min at room temperature. The reaction products were immediately resolved by non‐reducing sodium dodecyl sulfate–6 m urea–8% (w/v) polyacrylamide gel electrophoresis.

### Redox titration

The purified proteins were buffer exchanged with a PD‐10 column into a redox titration buffer (100 mm NaCl, 100 mm HEPES, pH 7.0). Aliquots of 2.5 µm Rre1, RpaA or RpaB proteins were equilibrated for 2 h at redox potentials ranging from −220 mV to −400 mV in a redox titration buffer at 22 °C. The different redox potentials were achieved by mixing different ratios of oxidised and reduced DTT at a final concentration of 2 mm. The redox‐titrated proteins were resolved on a non‐reducing SDS/urea/PAGE gel as before. The oxidised and reduced proteins migrated differently on the gel and their band intensities, as quantified by the Image Lab software (Bio‐Rad, Hercules, CA, USA), were used as a reporter for the redox titration. The equilibrium redox potential (*E*_h_) was calculated by the Nernst equation for a 2 electron redox reaction:

$$E_{h}=E_{0}+29.6\;\log\frac{\left[{\text{DTT}}_{\mathrm{ox}}\right]}{\left[{\text{DTT}}_{\mathrm{Red}}\right]}$$

where [DTT_ox_] and [DTT_red_] are molar concentrations of oxidised and reduced DTT respectively and *E*_0_ is the standard redox potential of DTT at pH 7.0 (−327 mV). The *E*_m_ of the redox‐responsive thiol group was calculated by fitting the data to the Nernst equation with the graphpad software.

### Size‐exclusion chromatography

To oxidize the recombinant proteins, 1.7 mg of Rre1, RpaA and RpaB were treated with 2 mm H_2_O_2_ and 1.7 mg of RppA with 2 mm diamide. The oxidant was removed by desalting with a MiniTrap G25 column. As previously stated, 2 mm DTT was used for reducing all target proteins. The oligomeric states of the oxidised and reduced proteins were determined with a Superdex S200 10/300GL Increase (GE Healthcare Life Sciences) size exclusion chromatography of 500 µL (0.85 mg) protein. The column was equilibrated with a buffer containing 100 mm HEPES (pH 8.0) and 100 mm NaCl for Rre1, RpaA and RppA or with 5% (v/v) glycerol, 300 mm NaCl and 100 mm HEPES (pH 8.0) for RpaB. For reduced proteins, the column was equilibrated with the same buffer but with 2 mm DTT added. The molecular weight was determined by a calibration curve generated using the following standard proteins of known molecular weight: thyroglobulin (669 kDa), apoferritin (443 kDa), β‐amylase (200 kDa), bovine serum albumin (66 kDa) and carbonic anhydrase (29 kDa). Blue dextran (2000 kDa) was used to determine the void volume (Vo).

## Results

### Rre1, RpaA, RpaB and RppA contain conserved cysteine residues as putative redox regulation target sites

**Fig. 1:**
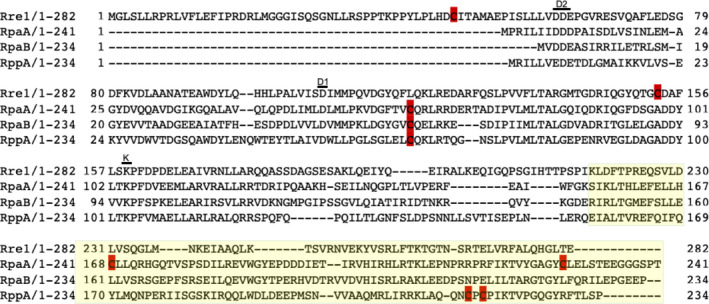
Sequence alignment of full‐length Synechocystis sp. PCC 6803 Rre1, RpaA, RpaB, and RppA response regulators. Putative redox‐active cysteines are shown in red. The divalent cation Mg^2+^‐binding motif “D2”, phosphorylation site “D1”, and the lysine (K) motifs are shown. The receiver domain is shaded in white, and the DNA binding domain in yellow.

To examine whether these response regulators contain conserved cysteine residues that could function as thiol modification sites, we aligned the amino acid sequence of Rre1, RpaA, RpaB and RppA. Fig. 1 and Fig. S1‐S4 show the presence of one or more conserved cysteine residues in all four response regulators. Cysteine residues are found in the receiver domain, which carries the aspartate phosphorylation site (D1 motif), as well as in the DNA‐binding domain. From the multiple sequence alignment of each protein orthologues (Fig. S1‐S4), it is apparent that the *Synechocystis* Rre1 contains two cysteine residues within its receiver domain. Of these, cysteine 153 is highly conserved in other cyanobacterial Rre1 sequences while the other cysteine is poorly conserved (Fig. S1). *Synechocystis* RpaA, RpaB and RppA contain a cysteine residue within their receiver domains and RpaA and RppA contain two additional cysteines within their DNA‐binding domains (Fig. 1). All three cysteines in RpaA are almost completely conserved (Fig. S2), and the single cysteine in RpaB is fully conserved (Fig. S3) as noted earlier [29]. For RppA, only the receiver domain cysteine 65 is fully conserved (Fig. S4).

### Redox conditions modulate the oligomeric state of Rre1, RpaA, RpaB and RppA

**Fig. 2:**
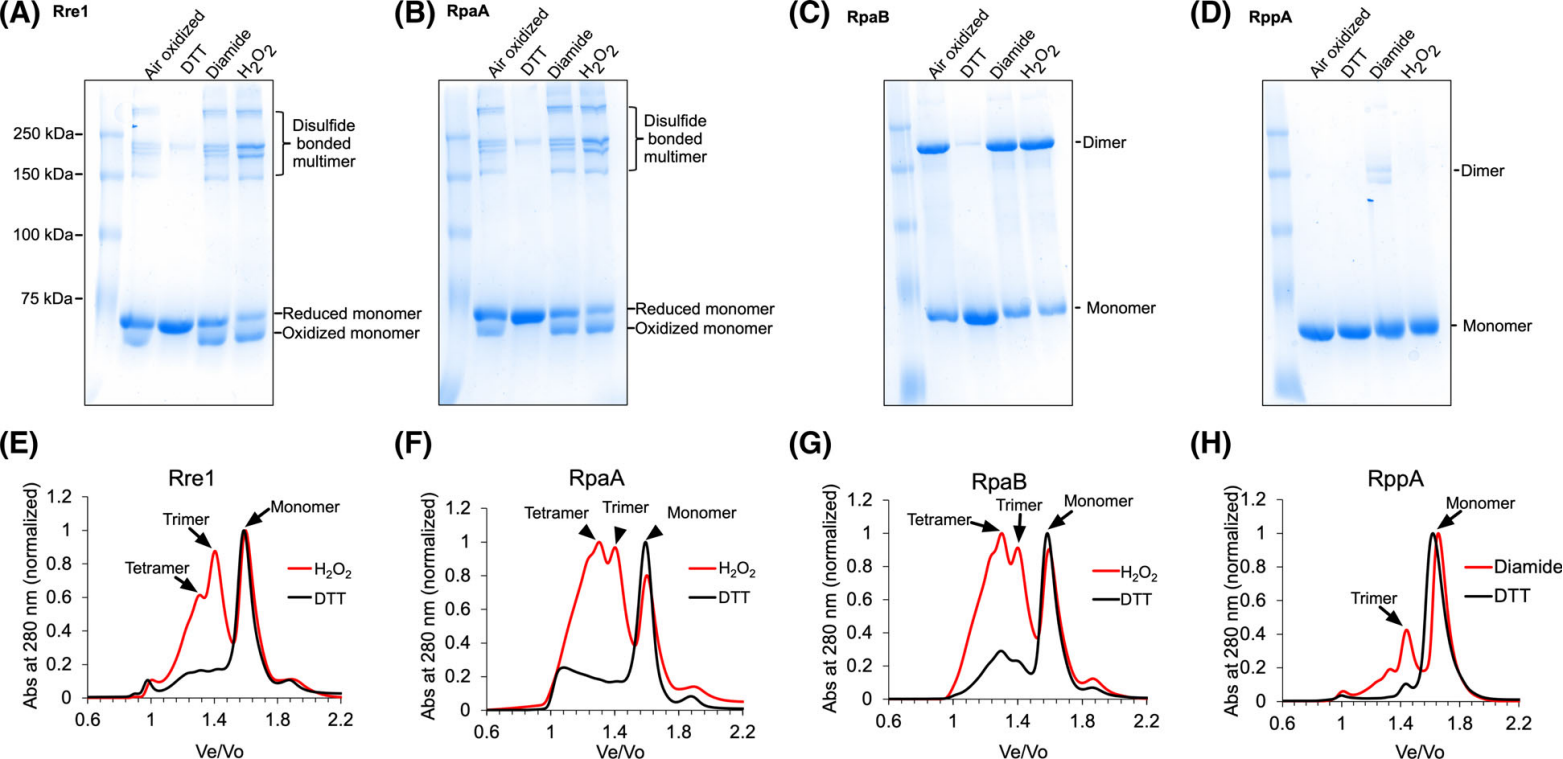
Effect of redox environment on the oligomeric state of Rre1, RpaA, RpaB, and RppA. (A–D) redox‐treated proteins as resolved by urea‐SDS/PAGE. (E–H) oligomeric state of response regulators as determined by size exclusion chromatography. Panels (E–H) correspond to samples oxidized with 2 mm H_2_O_2_ or reduced with 2 mm DTT, respectively.

Having identified putative redox‐responsive cysteine(s) in Rre1, RpaA, RpaB and RppA (Fig. 1), we examined whether redox treatments have any effect on the protein conformation or oligomeric state of these response regulators. Full‐length recombinant response regulator proteins were incubated with 2 mm cysteine‐specific oxidant diamide, or 2 mm H_2_O_2_, or 2 mm thiol reductant DTT and the redox‐treated proteins were then resolved on an 8% non‐reducing urea–SDS/PAGE gel. In the air‐exposed untreated and diamide‐, or H_2_O_2_‐treated Rre1 and RpaA samples, an oxidised fast‐migrating monomer protein band was present. This more compact form of the monomer presumably arose from an intramolecular disulfide bond and on the gel it migrates slightly below the reduced monomer protein. Several less intense bands above 180 kDa were also observed, with the higher‐order oligomers likely arising from multiple intermolecular disulfide bonds between three or four monomers (Fig. 2A,B). The addition of 2 mm DTT to the air‐oxidised samples resulted in monomerisation of all proteins (Fig. 2A,B). RpaB appears to dimerise *via* an intermolecular disulfide bond after being oxidised by the air or after treatment with diamide or H_2_O_2_ (Fig. 2C), and the dimer form can be converted to monomers after treatment of the air‐oxidised protein with 2 mm DTT (Fig. 2C). In contrast to these three response regulators, the air‐oxidised RppA protein migrated as a single monomer band. However, after treatment with diamide, two additional bands, corresponding to higher‐order oligomers, were apparent (Fig. 2D). To check whether the effect of diamide could be reversed, the diamide‐oxidised protein was first desalted to remove any residual diamide present. The desalted protein was subsequently treated with 2 mm DTT. DTT treatment indeed converts the higher‐order oligomers into monomers, supporting the role of cysteines in the redox control of RppA oligomeric state (Fig. 2D).

Size exclusion chromatography was used to probe further the oligomeric states of these response regulators in their native state under oxidising (H_2_O_2_) and reducing (DTT) conditions. Because the behaviour of proteins within the size exclusion gel matrix is more closely related to their hydrodynamic radius (Stokes radius, *R_s_*) than their molecular weight, obtaining an accurate molecular weight through this technique is difficult. For example, proteins with elongated shape might elute at a position that is significantly different from spherical proteins with the same molecular weight. Size exclusion chromatography can nevertheless provide an approximate molecular weight. To that end, we used the standard proteins listed in the Materials and Methods and Fig. S5 to calculate the approximate molecular weight of response regulators selected in this study. The oxidised form of Rre1 was eluted with apparent molecular weights of 104, 210 and 297 kDa on a Superdex S200 column. The theoretical molecular weight of a monomer of Rre1 tagged with MBP is 75 kDa. Thus, the eluted proteins likely correspond to monomer, trimer and tetramer of Rre1, respectively (Fig. 2E). Similarly, the oxidised RpaA (with a theoretical molecular weight of 71 kDa) and RpaB (with a theoretical molecular weight of 70 kDa) eluted as a monomer, trimer and tetramer with an apparent molecular weight of 103, 211 and 304 kDa for RpaA (Fig. 2F), and 109, 213 and 306 kDa for RpaB (Fig. 2G). Interestingly, size exclusion chromatography does not show any RpaB dimers in contrast to the non‐reducing gel (Fig. 2C). It is likely that the disulfide bonded‐dimers of RpaB, resolved in the protein gel (Fig. 2C), form tetramers (dimer of dimers) or other oligomers *via* noncysteine‐mediated interactions. Such oligomers will only be apparent in size exclusion chromatography but not in denaturing protein gel electrophoresis (Fig. 2C). The RppA (with a theoretical molecular weight of 70 kDa) eluted with apparent molecular weights of 84 and 184 kDa, corresponding to monomer and trimer, respectively (Fig. 2H). All four proteins eluted as a monomer in their reduced DTT‐treated forms (Fig. 2E‐H). The results obtained with size exclusion chromatography thus corroborate the oligomerisation patterns seen in non‐reducing urea–SDS/PAGE (Fig. 2A‐D).

### Midpoint potentials of redox‐active cysteines in Rre1, RpaA and RpaB suggest a regulatory role

**Fig. 3:**
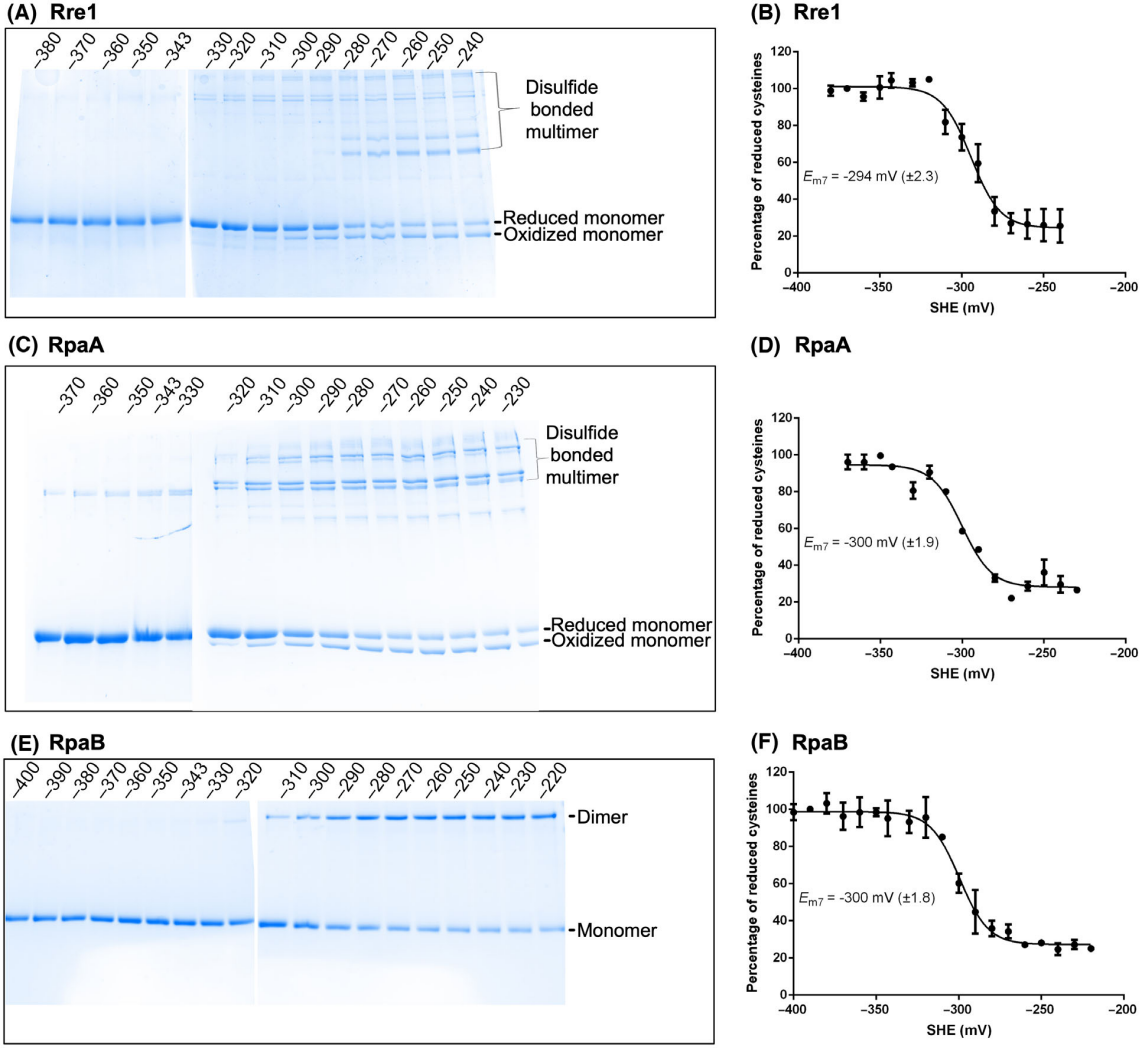
Redox titration of Rre1, RpaA, and RpaB. (A,C & E) Redox‐titrated proteins separated by nonreducing urea‐SDS/PAGE and stained with Coomassie brilliant blue. (B,D & F) Plots of redox titration. From the intensity of the reduced and oxidized band species, the midpoint potential E_m_ was computed by fitting the data to the Nernst equation. SHE, standard hydrogen electrode potential. Error bars represent ± SE from two replicate titrations.

Our results in Fig. 2 suggest that the response regulators studied here contain redox‐active disulfide linkages. Disulfide bonds might nevertheless have a structural rather than a regulatory role in these proteins. Disulfide bonds with structural roles, however, typically have a very negative midpoint potential as low as −470 mV [26] while cysteines with regulatory roles have a higher midpoint potential, ranging from −95 to −330 mV [27, 28, 30, 31, 32]. We therefore carried out a redox titration of the purified response regulators to determine their midpoint potentials. The results of redox titration for Rre1 (Fig. 3A,B), RpaA (Fig. 3C,D) and RpaB (Fig. 3E,F) at pH 7 are shown. Duplicate titrations gave an average *E_m_* value at pH 7 of −294 mV (± 2.5 mV) for Rre1, −300 mV (± 2 mV) for RpaA, and −300 mV (± 1.8 mV) for RpaB. Since all three *E_m7_* values are higher than −330 mV, the lowest potential reported for a regulatory disulfide [27, 28, 30, 31, 32], it is likely that the redox‐active cysteines of Rre1, RpaA and RpaB play a regulatory role. We did not perform a titration for RppA because only a small fraction of this protein could be oxidised with diamide.

### TrxA‐dependent reduction of Rre1, RpaA and RpaB

**Fig. 4:**
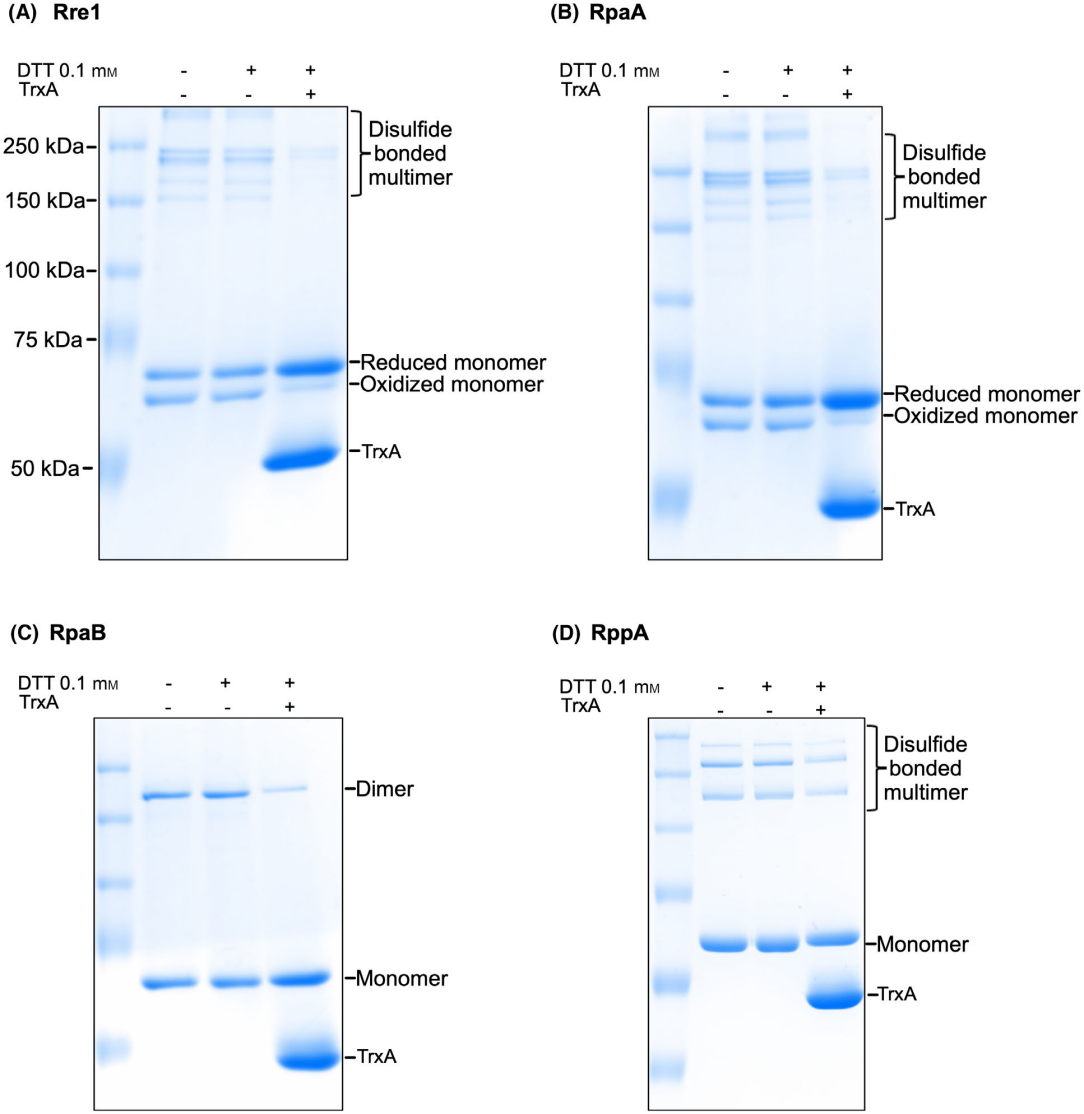
TrxA‐mediated reduction of the redox‐active cysteines of Rre1, RpaA, RpaB, and RppA. TrxA‐treated proteins as separated on a nonreducing urea‐SDS/PAGE gel and stained with Coomassie. + or − sign indicates the presence or absence of 0.1 mm DTT or 5 mm TrxA, respectively.

After establishing that Rre1, RpaA and RpaB contain redox‐active cysteines and have redox potentials that are consistent with regulatory thiols, we checked whether those cysteines are targets of the thioredoxin system. TrxA has been shown to interact with RpaA and RpaB and reduce them in vitro [29]. Although there is no direct evidence for Rre1 interaction with thioredoxin, its redox potential (Fig. 3B) suggests that it could also be a target of TrxA. Rre1 and RpaA proteins purified from *E. coli* mostly exist as a low molecular weight reduced and oxidised monomers with a small amount of oxidised higher‐order oligomers (Fig 4A,B). RpaB, as purified from bacteria, migrates as a reduced monomer and an oxidised dimer (Fig. 4C). Treatment of all three recombinant proteins with 0.1 mm DTT had little effect on their oligomeric state. However, incubation of Rre1 and RpaA with 5 mm TrxA in the presence of 0.1 mm DTT resulted in the conversion of the oxidised higher‐order oligomers or oxidised monomers into a reduced monomer; for RpaB, from dimer to monomer (Fig. 4A‐C). These results suggest that the redox‐active cysteines of Rre1, RpaA and RpaB could indeed be targets of TrxA regulation *in vivo*. Treatment of RppA with 0.1 mm DTT and TrxA had only a small effect on its aggregation state (Fig. 4D), indicating that it is unlikely to be a physiological TrxA substrate.

## Discussion

The *Synechocystis* genome contains six thioredoxin genes. These thioredoxin proteins are likely reduced by two separate enzymes: ferredoxin‐thioredoxin reductase (FTR) and NADPH‐thioredoxin reductase (NTR), which receive reducing equivalents from ferredoxin and NADPH, respectively. Through the thiol‐disulfide interconversion of target proteins, the thioredoxin system connects the redox state of photosynthetic electron transport with metabolic regulation of both light and dark reactions of photosynthesis. Light plays a critical role in fine‐tuning gene expression in cyanobacteria. High light treatment or moving the cyanobacterial cells to darkness promotes the downregulation of several photosystem genes, most notably genes encoding photosystem I (PSI) and phycobilisome subunits [33]. Genes encoding photosystem II (PSII) components respond in the opposite direction as they become upregulated in high light conditions. Interestingly, the expression profiles of *FTR*, *trxA* and *trxB* genes mimic that of PSI and phycobilisome genes. Notably, these three genes are further shown to be regulated by the redox state of the PQ pool [33].

Given previous reports of interaction between the ferredoxin‐thioredoxin system and Rre1, RpaA and RpaB, the current study sought to characterise whether these response regulators indeed contain thioredoxin‐targeted thiol‐disulfide redox switches for the regulation of their oligomeric state and activity. All four response regulators contain at least one highly conserved cysteine residue within their receiver domain where phosphorylation takes place (Fig. 1, Fig. S1‐S4). This raises two interesting but not mutually exclusive possibilities: the redox‐responsive cysteines might control the accessibility of the key aspartate phosphorylation site for the sensor kinase and or the oligomeric state of the response regulators. Under oxidising conditions, Rre1 and RpaA proteins formed an intramolecular disulfide‐bonded monomer and intermolecular disulfide‐bonded higher‐order oligomers. RpaB under oxidising conditions formed a dimer *via* an intermolecular disulfide bond as revealed by the non‐reducing urea–SDS/PAGE (Fig. 2A‐C). The oxidised RppA had two high molecular weight bands, corresponding to a trimer and probably another higher‐order oligomer (Fig. 2D).

Although response regulators usually have a single DNA‐binding domain, the active state of many response regulators is dimeric. This allows them to recognise tandem or inverted DNA repeat elements for transcriptional regulation. For FixJ, Spo0A, and OmpR/PhoB subfamily of response regulators, phosphorylation induces conformational changes in the α4‐β5‐α5 face of the receiver domain. This promotes dimerisation of the response regulators *via* their receiver domains. Phosphorylation‐mediated dimerisation thus enhances DNA‐binding activity and ultimately the extent of transcriptional regulation by response regulators [34]. An intriguing possibility is that thiol modification represents an independent or synergistic (with phosphorylation) mechanism that promotes dimerisation or oligomerisation of response regulators. Our analyses of Rre1, RpaA, RpaB and RppA by non‐reducing gel electrophoresis and size exclusion chromatography are consistent with this possibility. The DTT‐reduced proteins eluted as monomers in size exclusion chromatography (Fig. 2E‐H), which is consistent with results obtained by non‐reducing urea–SDS/PAGE (Fig. 2A‐D). Under oxidising condition of air, all proteins were converted into higher‐order oligomers. Expectedly, stronger oxidising agents such as H_2_O_2_ or diamide enhanced oligomerisation (Fig. 2). It is possible that the disulfide‐mediated oligomerisation of these regulators is sufficient for enhancing their DNA‐binding activity, or the thiol modification likely acts synergistic with phosphorylation. Whether the more compact internally disulfide‐bonded Rre1 and RpaA monomers are functionally relevant also remains to be determined (Fig. 2A,B). Rre1 has recently been shown to be phosphorylated *in vivo* under reduced PQ pool condition during thermal stress, possibly *via* its cognate sensor kinase Hik2. Phosphorylation of Rre1 leads to *heat shock protein A* (*hspA*) gene transcript accumulation under the same condition [11]. Interestingly, Bairagi et al. [11] also find some *hspA* transcript accumulation even in the absence of Rre1 phosphorylation, suggesting a phosphorylation‐independent regulation of the *hspA* gene. The oxidation‐induced oligomerisation that we report here might represent such a phosphorylation‐independent pathway for regulation of Rre1 activity.

What is the nature of the *in vivo* redox signals that likely act upon the thiol redox switches of Rre1, RpaA and RpaB? Redox regulation of transcription factors is a widely recognised fundamental regulatory mechanism for gene expression. The Fd‐Trx system plays a crucial role in this process. RpaA, RpaB and Rre1 were shown to interact with TrxA [29, 35] or Fd [36]. The RpaA–Fd interaction was found to be strongest under reducing conditions, indicating that the interaction occurs in light and might serve as a diurnal input to downregulate RpaA transcriptional activity. The midpoint potentials of redox‐active cysteines of Rre1, RpaA and RpaB at pH 7 are −294, −300, and −300 mV, respectively (Fig. 3). Cyanobacterial and plant thioredoxins have an *E_m7_* value of around −300 mV [37, 38]. This redox potential of thioredoxin is closer to the *E_m_* of Rre1, RpaA and RpaB (Fig. 3), making their TrxA‐mediated reduction thermodynamically favourable. Indeed, in this study, and in [29] TrxA was able to reduce the oxidised cysteines of different response regulators (Fig. 4). Thus, m‐type thioredoxin might thus downregulate the activity of these response regulators *in vivo*. An additional physiologically relevant activating redox signal is likely to be H_2_O_2_ as growth of cyanobacterial cells in ambient air at 40 °C induces oxidative stress in the form of H_2_O_2_ [39]. The elevated H_2_O_2_ at high temperature could be an important regulatory signal that mediates the oligomerisation of Rre1, RpaA and RpaB. Reducing conditions *via* the Trx system might reverse the effect of H_2_O_2_. Taken together, our results suggest that thiol redox regulation is likely to be an important mechanism in the regulation of the activity of these cyanobacterial response regulators independently of, or synergistic with, their cognate sensor kinase‐mediated transphosphorylation.

## Conflict of interest

The authors declare no conflict of interest.

## Author contributions

IMI, SJLR, WAC, CJH and SP conceived, conceptualised and wrote the manuscript. IMI curated data and devised methodology. All authors have read and agreed to the published version of the manuscript.

## Supporting information


**Fig**. **S1**. Conserved cysteine of Rre1.
**Fig. S2.** Conserved cysteines of RpaA.
**Fig. S3.** Conserved cysteine of RpaB.
**Fig. S4.** Conserved cysteines of RppA.
**Fig. S5.** Protein standard curve.

**Data S1**. Datasets used to plot Fig. 2E,F and Fig. 3B,D,F.

## Data Availability

The dataset generated and analysed in the current study is available as Supplementary Data S1. All other data (if any) are available upon request.
