# Supplementary material for: Thiol redox switches regulate the oligomeric state of cyanobacterial Rre1, RpaA and RpaB response regulators
Source: FEBS Lett. 2022 Apr 11;596(12):1533–43. doi: 10.1002/1873-3468.14340 (PMC9321951; doi:10.1002/1873-3468.14340)
Supplement: Supplementary file 1 — Fig. S1. Conserved cysteine of Rre1. Fig. S2. Conserved cysteines of RpaA. Fig. S3. Conserved cysteine of RpaB. Fig. S4. Conserved cysteines of RppA. Fig. S5. Protein standard curve. [file FEB2-596-1533-s002.pdf]

Supplementary Figures

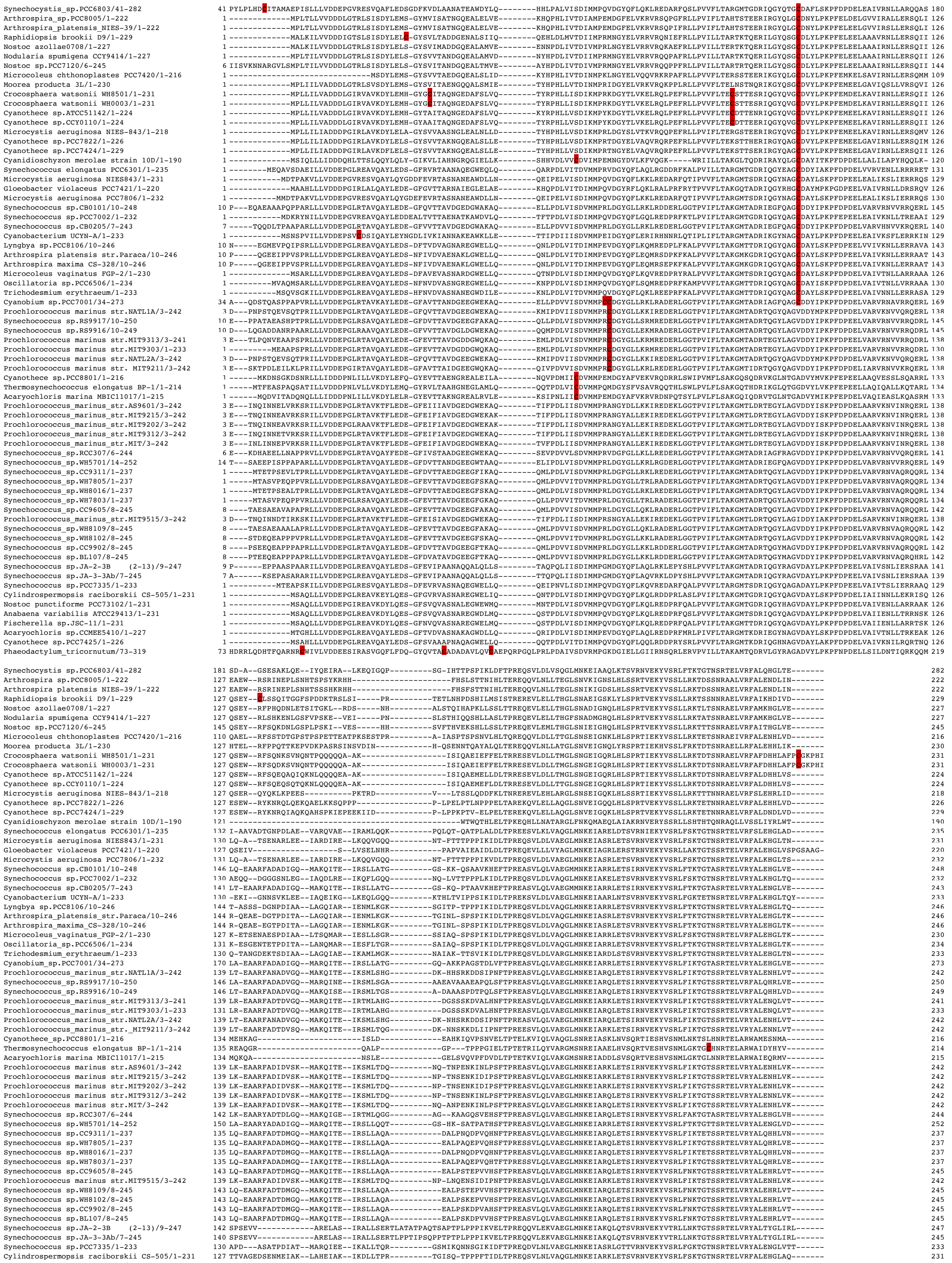

Fig. S1. **Conserved cysteine of Rre1**. A multiple sequence alignment of cyanobacterial Rre1 orthologues showing the cysteine residues highlighted in red.

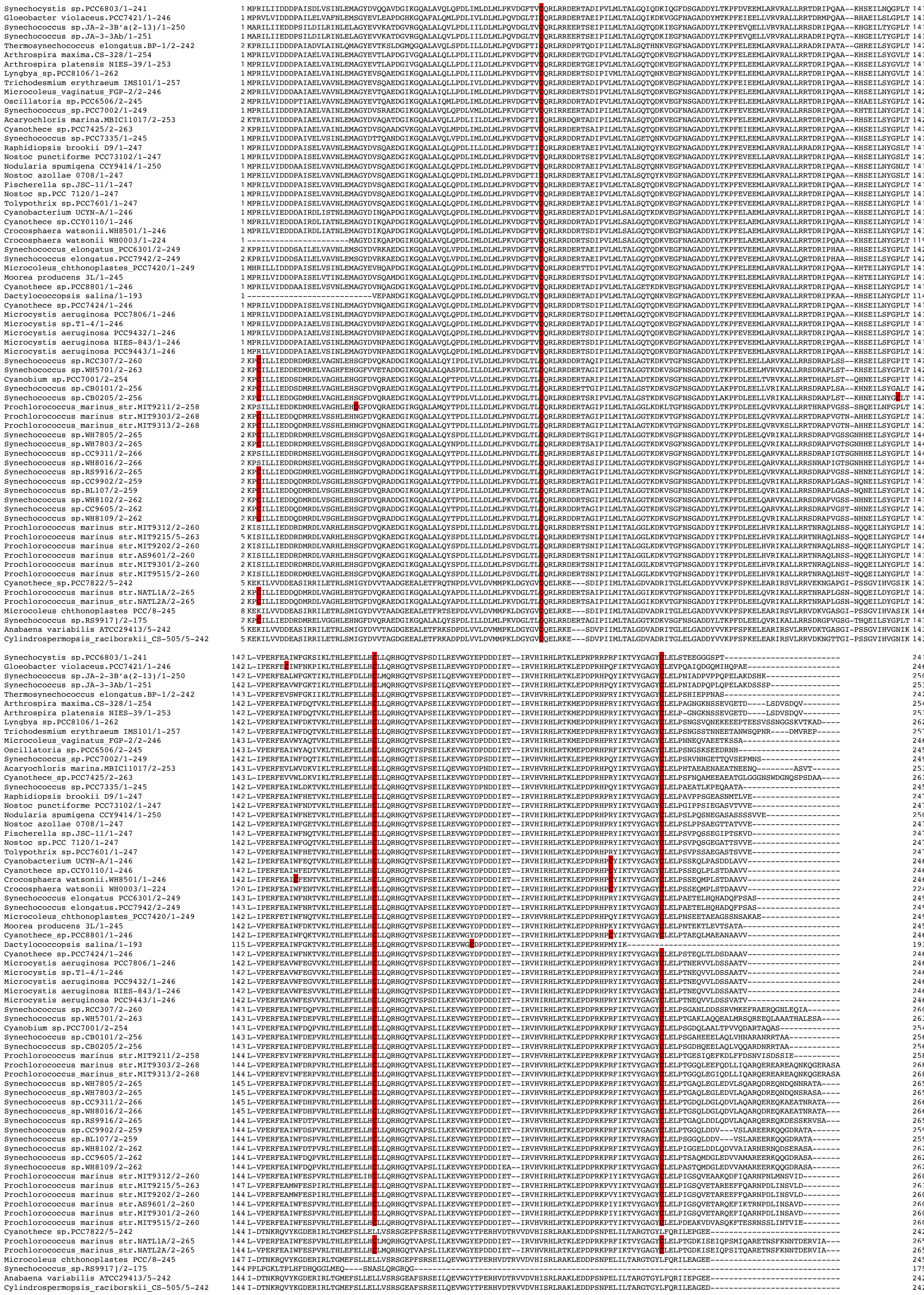

Fig. S2. **Conserved cysteines of RpaA.** A multiple sequence alignment of cyanobacterial RpaA orthologues showing the cysteine residues highlighted in red.

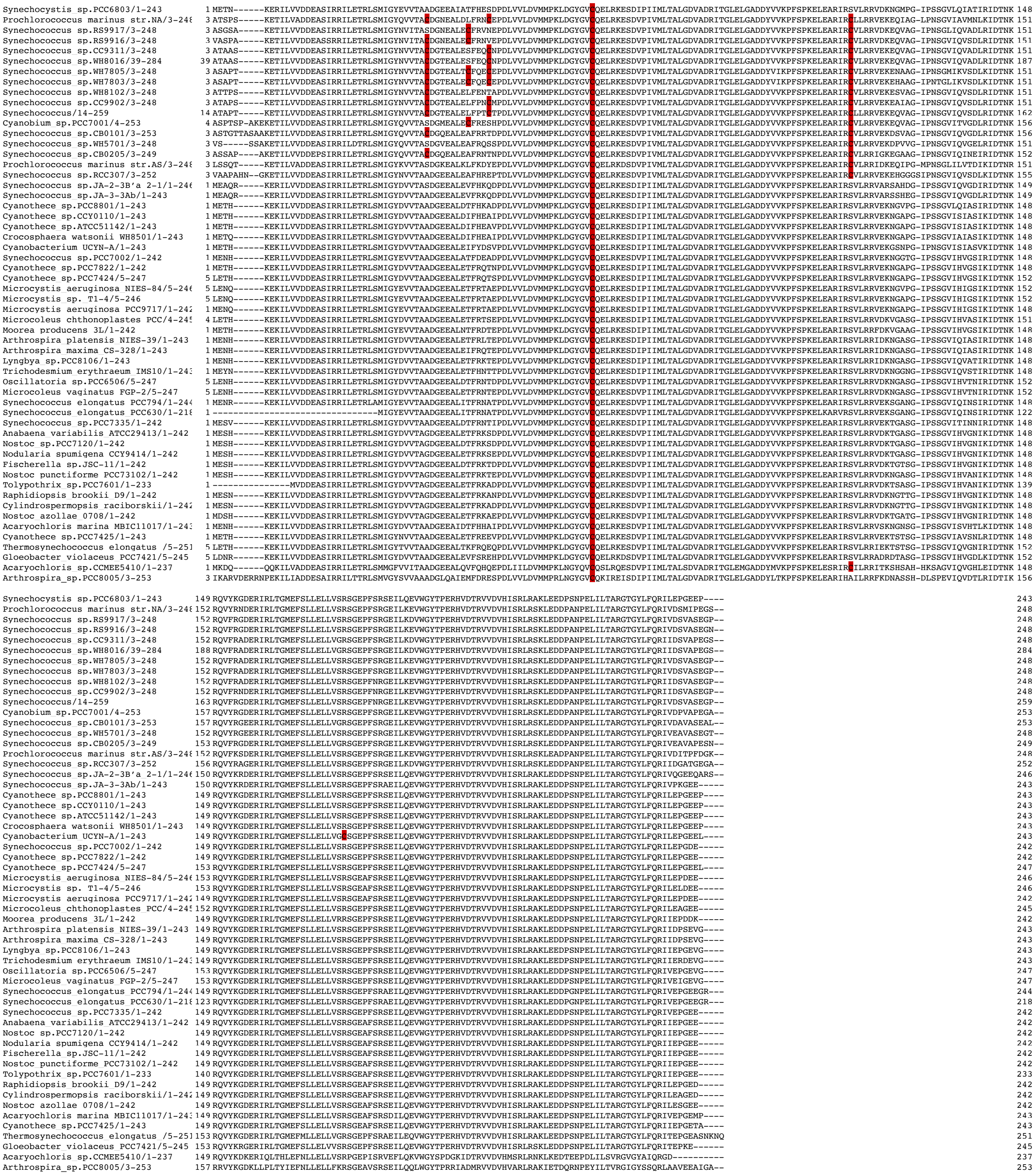

Fig. S3. **Conserved cysteines of RpaB.** A multiple sequence alignment of cyanobacterial RpaB orthologues showing the cysteine residues highlighted in red.

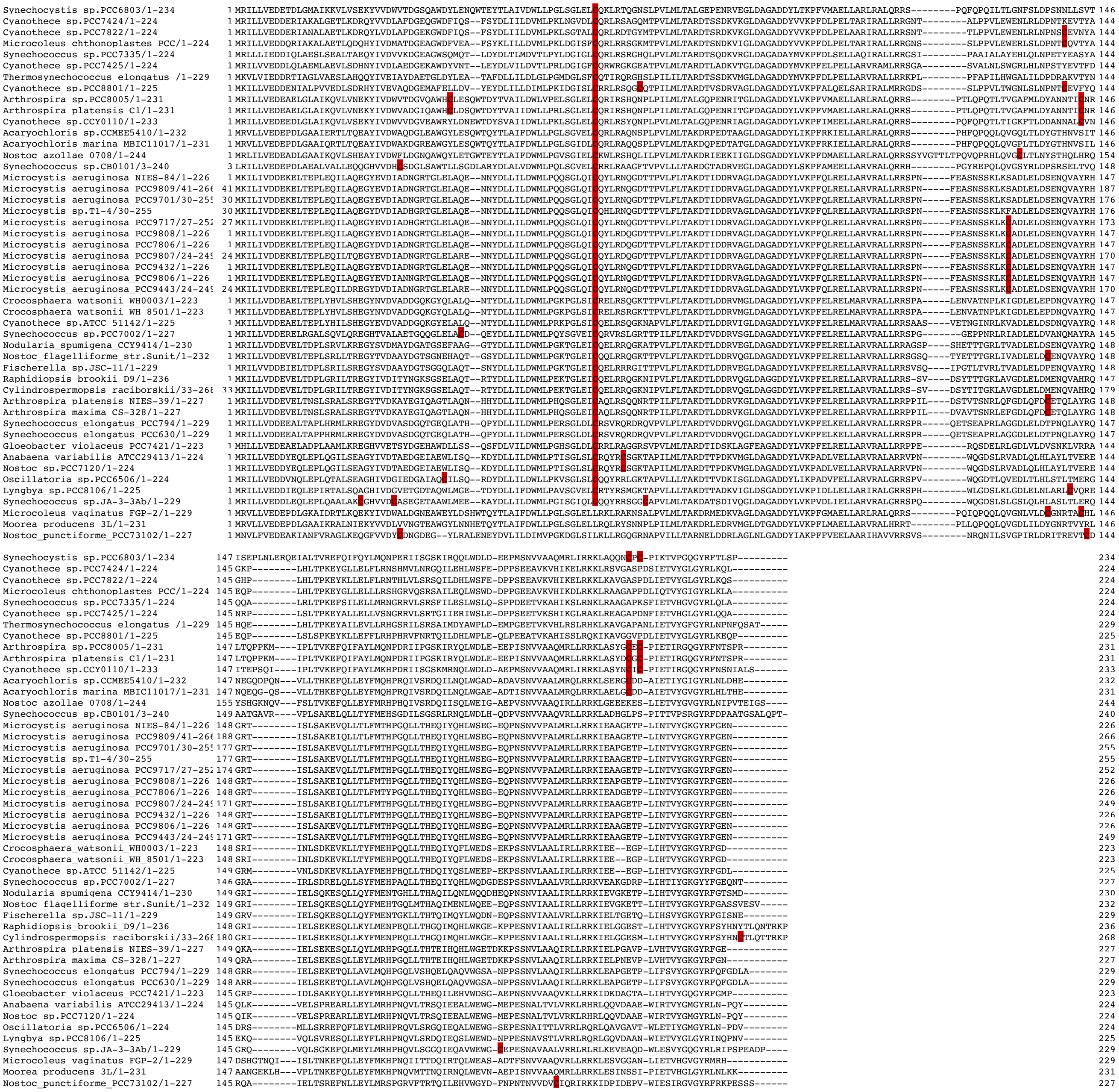

Fig. S4. **Conserved cysteine of RppA.** A multiple sequence alignment of cyanobacterial RppA orthologues showing the cysteine residues highlighted in red.

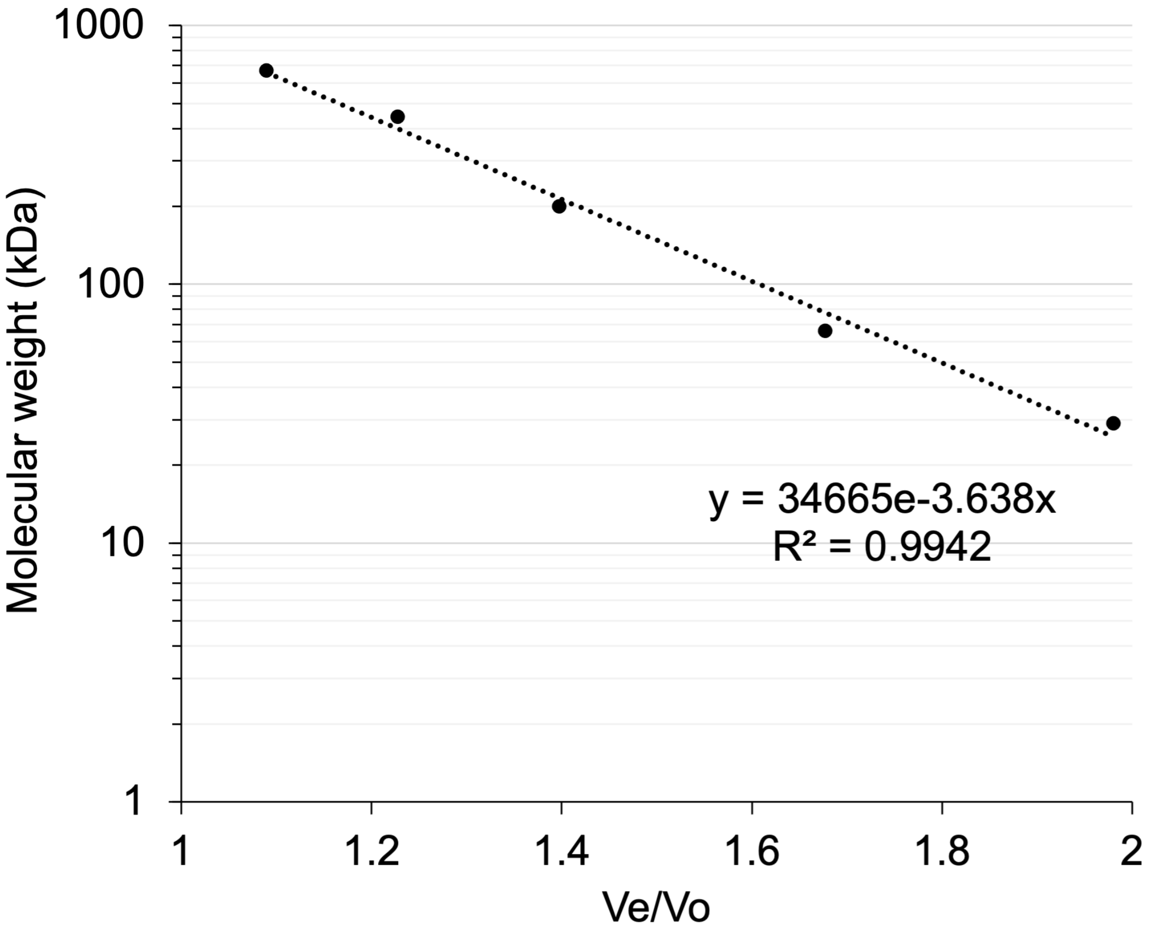

Fig. S5. Calibration curves of the Superdex 200 column. Calibration curve was obtained using standard proteins of known molecular mass: thyroglobulin (669 kDa), apoferritin (443 kDa), β-amylase (200 kDa), bovine serum albumin (66 kDa) and carbonic anhydrase (29 kDa). Blue dextran (2000 kDa) was used to determine the void volume (Vo). Ve is the effluent volume. On the y-axis the base-ten logarithm of the protein molecular mass is shown and, on the x-axis, Ve/V_0.
